# Prevalence of *TECTA* mutation in patients with mid-frequency sensorineural hearing loss

**DOI:** 10.1186/s13023-017-0708-z

**Published:** 2017-09-25

**Authors:** Nobuko Yamamoto, Hideki Mutai, Kazunori Namba, Noriko Morita, Shin Masuda, Yasuyuki Nishi, Atsuko Nakano, Sawako Masuda, Masato Fujioka, Kimitaka Kaga, Kaoru Ogawa, Tatsuo Matsunaga

**Affiliations:** 1 Department of Otolaryngology, National Hospital Organization Tokyo Medical Center, 2-5-1 Higashigaoka, Meguro, Tokyo, 152-8902 Japan; 2 Division of Hearing and Balance Research, National Institute of Sensory Organs, National Hospital Organization Tokyo Medical Center, 2-5-1 Higashigaoka, Meguro, Tokyo, 152-8902 Japan; 3 Department of Otolaryngology, Head and Neck Surgery, Keio University School of Medicine, 35 Shinanomachi, Shinjuku, Tokyo, 160-8582 Japan; 4 Department of Otolaryngology, Teikyo University School of Medicine, 2-11-1 Kaga, Itabashi, Tokyo, 173-8606 Japan; 5 Department of Pediatric Rehabilitation, Hiroshima Prefectural Hospital, 1-5-54 Ujina-Kanda, Minami, Hiroshima, 734-8530 Japan; 6 Department of Otolaryngology, National Hospital Organization Kure Medical Center, 3-1 Aoyama, Kure, Hiroshima, 737-0023 Japan; 7 Division of Otorhinolaryngology, Chiba Children’s Hospital, 579-1 Heta, Midori, Chiba, 266-0007 Japan; 8 Department of Otorhinolaryngology, National Mie Hospital, 357 Osato-Kubota, Tsu, Mie 514-0125 Japan

**Keywords:** DFNA8/12, DFNB21, TECTA, Mid-frequency hearing loss

## Abstract

**Background:**

To date, 102 genes have been reported as responsible for non-syndromic hearing loss, some of which are associated with specific audiogram features. Four genes have been reported as causative for mid-frequency sensorineural hearing loss (MFSNHL), among which *TECTA* is the most frequently reported; however, the prevalence of *TECTA* mutations is unknown. To elucidate the prevalence of *TECTA* mutation in MFSNHL and clarify genotype-phenotype correlations, we analyzed the genetic and clinical features of patients with MFSNHL.

**Methods:**

Subjects with bilateral non-syndromic hearing loss were prescreened for *GJB2* and m.1555A > G and m.3243A > G mitochondrial DNA mutations, and patients with inner ear malformations were excluded. We selected MFSNHL patients whose audiograms met the U-shaped criterion proposed by the GENDEAF study group, along with those with shallow U-shaped audiograms, for *TECTA* analysis. All *TECTA* exons were analyzed by Sanger sequencing. Novel missense variants were classified as possibly pathogenic, non-pathogenic, and variants of uncertain significance, based on genetic data. To evaluate novel possibly pathogenic variants, we predicted changes in protein structure by molecular modeling.

**Results:**

Pathogenic and possibly pathogenic variants of *TECTA* were found in 4 (6.0%) of 67 patients with MFSNHL. In patients with U-shaped audiograms, none (0%) of 21 had pathogenic or possibly pathogenic variants. In patients with shallow U-shaped audiograms, four (8.7%) of 46 had pathogenic or possibly pathogenic variants. Two novel possibly pathogenic variants were identified and two previously reported mutations were considered as variant of unknown significance. The clinical features of patients with pathogenic and possibly pathogenic variants were consistent with those in previous studies. Pathogenic or possibly pathogenic variants were identified in 3 of 23 families (13.0%) which have the family histories compatible with autosomal dominant and 1 of 44 families (2.3%) which have the family histories compatible with sporadic or autosomal recessive.

**Conclusions:**

*TECTA* mutations were identified in 6.0% of MFSNHL. These mutations were more frequent in patients with shallow U-shaped audiograms than those with U-shaped audiograms, and in families which have the family histories compatible with autosomal dominant than those with the family histories compatible with sporadic or autosomal recessive.

**Electronic supplementary material:**

The online version of this article (10.1186/s13023-017-0708-z) contains supplementary material, which is available to authorized users.

## Background

Sensorineural hearing loss (SNHL) is one of the most common sensory disorders in humans and its onset can be influenced by numerous environmental and genetic factors. Approximately 1 in 1000 newborns has congenital bilateral SNHL and around half of these have underlying genetic causes [1]. To date,102 genes have been reported as responsible for non-syndromic hearing loss [2], some of which are associated with specific audiogram features. There are four genes reported to cause mid-frequency hearing loss: *EYA4* (DFNA10), *TECTA* (DFNA8/12, DFNB21), *COL11A2* (DFNA13), and *CCDC50* (DFNA44) [3]. Among these four genes, mutations in *TECTA* are most frequently reported [4–7], and autosomal dominant (AD) *TECTA* mutations (DFNA8/12) account for 2.9–4% of all autosomal dominant non-syndromic sensorineural hearing loss (ADNSHL) [4, 7].

**Fig. 1:**
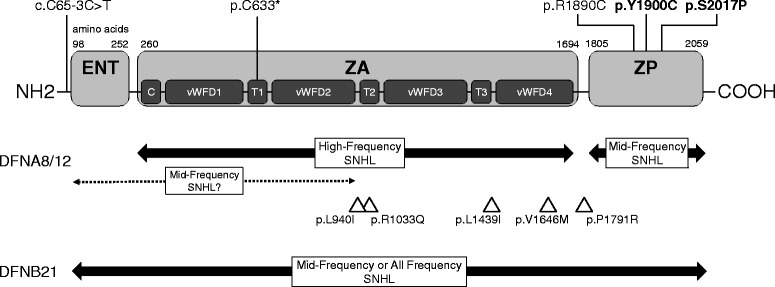
Domains of the *TECTA* and genotype-phenotype correlation of mutations. Pathogenic and possibly pathogenic variants found in this study are shown at the top of the scheme and the novel variants are highlighted in bold. Under the scheme of the domains, reported genotype-phenotype correlations for DFNA8/12 and DFNB21 are shown by bold lines with arrows for established phenotypes and a dotted line with arrows for proposed phenotypes. The triangles indicate the positions of the VUS found in this study. ENT, entactin-G1-like domain; ZA, zonadhesin-like domain; C, von Willebrand factor C domain; vWFD, von Willebrand factor D domain; T, trypsin inhibitor-like domain; ZP, zona pellucida domain; SNHL, sensorineural hearing loss

The *TECTA* gene is located on chromosome 11q22–q24, contains 23 exons, encodes 2155 amino acids, and generates the protein, α-tectorin, a non-collagenous component of the tectorial membrane [8]. The α-tectorin protein is composed of three distinct modules (Fig. 1) [9]: an entactin-G1-like domain (ENT); a zonadhesin-like (ZA) domain, comprising one von Willebrand factor type C repeat, four von Willebrand factor type D repeats, and three trypsin inhibitor-like repeats; and a zona pellucida (ZP) domain. Both AD and autosomal recessive (AR) inheritance patterns have been reported for mutations in *TECTA* (DFNA8/12, OMIM # 601543; DFNB21, OMIM # 603629 [8, 10]). Mouse models of deafness due to *TECTA* mutations exhibit deformation of the tectorial membrane [11–13]; however, the detailed mechanism by which these mutations cause mid-frequency hearing loss remains unknown.

Genotype-phenotype correlations for *TECTA* mutations are shown in Fig. 1. In DFNB21, pathogenic variants result in premature protein truncation, all showing prelingual, moderate to severe hearing loss, and flat or U-shaped audiograms, regardless of the region of mutation [10, 14, 15]. In DFNA8/12, pathogenic variants are generally missense, with the audiogram dependent on the region of the variant: high-frequency SNHL (HFSNHL) associated with mutations of the ZA domain; and mid-frequency SNHL (MFSNHL) associated with ZP domain alterations [16, 17]. Recently, association with MFSNHL were reported not only in the ZP domain mutations but also in the ENT domain and N-terminal region of the ZA domain [4].

Although MFSNHL is common among patients with *TECTA* mutations, the prevalence of *TECTA* mutations in this group is unknown. To elucidate the prevalence of *TECTA* mutation in MFSNHL and clarify genotype-phenotype correlations, we analyzed the genetic and clinical features of patients presenting with MFSNHL.

## Methods

### Subjects

Subjects with MFSNHL were selected from patients who had undergone genetic tests at our institute from 2002 to 2016 and tested for *TECTA* mutations. This study was approved by the Institutional Review Boards of our hospital and each of the participating facilities, and conducted in accordance with the Declaration of Helsinki. All subjects or their parents (if the subject was less than 20 years old) provided written informed consent for participation in this project.

The criteria proposed by the GENDEAF study group published in Hereditary Hearing Loss Homepage [2] were used to categorize hearing-loss levels as follows: mild, 20–40 dB HL; moderate, 41–70 dB HL; severe, 71–95 dB HL; profound, >95 dB HL (better hearing ear, averaged over 0.5, 1, 2, and 4 kHz). The following criteria were used to define hearing loss progression: progressive, deterioration of >15 dB HL on average over the frequencies 0.5, 1, and 2 kHz within a 10-year period [2]. We judged the progression only in cases we could follow up the audiograms for more than 10 years.

**Fig. 2:**
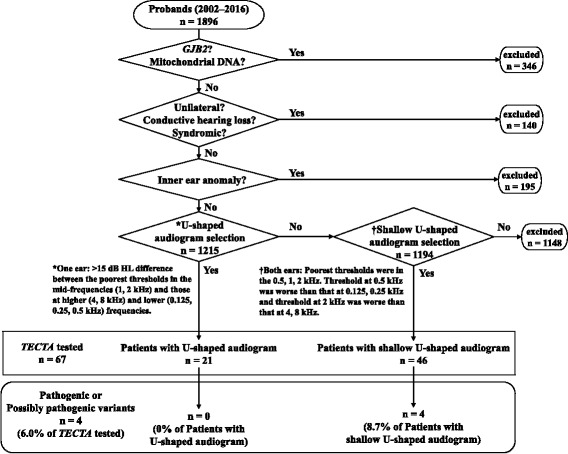
Flowchart of patient selection for *TECTA* analysis and the results. The selection of patients started with 1896 patients and patients with *GJB2* and mitochondrial mutations, and unilateral, conductive, and syndromic hearing loss, and inner ear anomaly were excluded. Then, the 1215 patients were subjected to U-shaped audiogram selection and 21 patients had U-shaped audiogram. Next, the 1194 patients who did not meet the criterion for U-shaped audiogram were subjected to Shallow U-shaped audiogram selection and 46 patients had shallow U-shaped audiogram. For the 67 patients (21 patients with U-shaped and 46 patients with shallow U-shaped) were tested for *TECTA* mutation. Finally, 4 patients (none with U-shaped and 4 patients with shallow U-shaped) had pathogenic or possibly pathogenic variants

The process for selecting patients for *TECTA* gene mutation testing is illustrated in Fig. 2. First, patients with hearing loss due to *GJB2*, or mitochondrial m.1555A > G or m.3243A > G, mutations were excluded based on previous test results. Next, patients with bilateral non-syndromic SNHL were selected, followed by exclusion of patients with inner ear malformations by CT or MRI, if performed. CT or MRI was performed in 1153 patients among 1410 bilateral non-syndromic SNHL patients. Next, patients were selected based on the mid-frequency U-shaped criterion proposed by the GENDEAF study group [2]: >15 dB HL difference between the poorest threshold at mid-frequencies (1, 2 kHz) and those at higher (4, 8 kHz) and lower (0.125, 0.25, 0.5 kHz) frequencies. Since many patients with shallow U-shaped audiograms did not meet this requirement, an additional new criterion was developed to include such patients: the poorest thresholds were identified at 0.5, 1, or 2 kHz, and the criterion was that the threshold at 0.5 kHz was worse than those at 0.125 and 0.25 kHz, and the threshold at 2 kHz was worse than those at 4 and 8 kHz; patients who did not meet the U-shaped criterion, but met this criterion for a shallow U-shaped audiogram, were also selected for *TECTA* analysis.

### Genetic analysis

Genomic DNA samples were extracted from the peripheral blood of patients using a DNA extraction kit Genomix (Biologica, Japan). The methods of screening for *GJB2* and the mitochondrial m.1555A > G and m.3243A > G mutations were shown in Additional file 1. For *TECTA* analysis, the primers described in Additional file 2 were used to amplify all exons of *TECTA* by PCR [17]. The PCR conditions were as follows: 5 min denaturation at 95 °C; 35 cycles of 95 °C for 1 min, 58 °C for 1 min, and 72 °C for 2 min; followed by 72 °C for 2 min, and ending with a holding period at 4 °C. PCR products were purified and subjected to Sanger sequencing. Analysis was performed using SeqScape ver3.0 Software (Applied Biosystems), by comparison with the NCBI human reference sequence (GRCh37.p13). Frameshift, splice site (splice site within ±2 nucleotides), and nonsense mutations were judged pathogenic. For changes in splice sites within 10 bp of exon-intron boundaries, NNSPLICE (0.9 version) [18] was used to predict the effect on splicing.

The reported *TECTA* missense variants were judged as pathogenic when the variants co-segregate with the phenotypes in family members, and judged as variants of uncertain significance (VUS) when the variants did not co-segregate with the phenotypes. Novel missense variants were judged as possibly pathogenic if they met all of the following criteria: 1) non-synonymous; 2) minor allele frequency < 1% in all public databases, including 1000 GENOMES [19], NHLBI Exome Sequencing Project (ESP6500) [20], Exome Aggregation Consortium (ExAC) [21], Human Genetic Variation Database (HGVD) [22], and integrative Japanese Genome Variation Database (iJGVD) [23]; 3) high amino acid conservation (>90% in primates and mammals, >50% in vertebrates) among up to 12 primate, 50 mammal, and 38 vertebrate species (Additional file 3) using the UCSC conservation tool [24]; 4) consistency with phenotypes confirmed by hearing tests in family members. Variants that did not satisfy criteria 1)–3) were judged as non-pathogenic. As patients with *TECTA* mutations often show mild to moderate hearing loss from childhood, individuals may not be aware of their hearing loss; therefore, variants that met criteria 1)–3), but failed criterion 4), were considered VUS. Furthermore, the effect of missense mutation on the function of the TECTA protein was predicted by computer analysis using PolyPhen-2 [25] and Protein Variation Effect Analyzer (PROVEAN v1.1.5) [26].

### Molecular modeling

To evaluate novel possibly pathogenic variants (p.Y1900C and p.S2017P), changes in the TECTA protein structure were predicted by molecular modeling. The tyrosine at residue 1900 of the TECTA protein is located in the N-terminal part of the ZP domain (ZP-N), while the serine at residue 2017 is located in the C-terminus of the ZP domain (ZP-C). As the structure of the TECTA ZP domain is unknown, the human ZP domain structure was predicted using the crystal structure of chicken ZP3 (PDB ID: 3NK3, chain A), which has a high amino acid homology to human ZP domains, as a template, using SWISS-MODEL [27–29]. The TECTA protein can form dimers through binding sites in the ZP-N domain, and the amino acid sequence of this domain is important for dimer formation [30, 31]. Therefore, as the p.Y1900C mutation may affect ZP-N dimerization, additional analysis of the effect of this variant on dimerization was performed. Since the dimeric structure of the ZP3 protein is unknown, a predicted structure of the human TECTA dimer was generated, using the predicted human ZP domain structure and the dimeric human uromodulin protein structure (PDB ID: 4WRN, chain A, chain B), which has high homology with the human ZP domain, as a template, using the display software, UCSF Chimera 1.11 [30, 32], and the influence of p.Y1900C mutation in this context was predicted.

## Results

### *TECTA* variants in patients with MFSNHL

As shown in the patient selection and exclusion diagram (Fig. 2), 1215 patients received audiogram selection from 1896 probands. There were 21 patients with U-shaped audiograms meeting the GENDEAF criterion in one or both ears (1.7% of patients who received U-shaped audiogram selection) and 46 with shallow U-shaped audiograms (3.9% of patients who were excluded by U-shaped audiogram selection). Family histories of these 67 patients with U-shaped or shallow U-shaped audiograms were compatible with AD in 23 patients (34.3%), AR in 10 patients (14.9%), and sporadic in 34 patients (50.7%). Gene analysis identified pathogenic and possibly pathogenic variants in 4 patients (6.0%) of the 67 patients. In patients with U-shaped audiograms, none had pathogenic or possibly pathogenic variants, one patient (4.8%) had VUS, and 20 patients (95.2%) had non-pathogenic variants or no variants (Additional file 4). In patients with shallow U-shaped audiograms, two patients (4.3%) had pathogenic variants, the other two patients (4.3%) had possibly pathogenic variants, five patients (10.9%) had VUS, and 37 patients (80.4%) had non-pathogenic variants or no variants. Pathogenic or possibly pathogenic variants were identified in 3 of 23 families (13.0%) which have the family histories compatible with AD and 1 of 44 families (2.3%) which have the family histories compatible with sporadic or AR.

A list of pathogenic variants, possibly pathogenic variants, and VUS found in this study is presented in Table 1, and a list of non-pathogenic variants is provided in Additional file 5. The DNA chromatograms of pathogenic variants, possibly pathogenic variants, and VUS are provided in Additional file 6. Three pathogenic variants (p.R1890C, p.C633*, and c.65-3C > T) have previously been reported. Two novel variants, p.Y1900C and p.S2017P, identified in this study met criteria 1)–4) (See Methods), and were predicted to have high pathogenicity by PolyPhen-2 and PROVEAN. Thus, they were considered possibly pathogenic variants.

**Table 1:** Pathogenic or possibly pathogenic variants and variants of uncertain significance in *TECTA* identified in this study

Type of variant	Location	Amino acid change	Nucleotide change	Genomic Position(Chr11)	Domain	Evolutionary Conservation^a^	Allele frequency					PolyPhen2 (Hum Var) score	PROVEAN score	Family	Reference
							1000 Genome	ESP 6500	ExAC	HGVD	iJGVD				
Pathogenic Variants															
Missense	Exon 18	p.R1890C	c.5668C > T	121038844	ZP	12/12, 50/50, 22/36	0	0	0	0	0	Probably damaging (0.963)	Neutral (−1.914)	4	[4, 16]
Nonsense	Exon 8	p.C633*	c.1899C > A	120998585	ZA (TIL1)	11/12, 47/47, 36/37	0	0	0	0	0	–	–	5	[39]
Splice site	IVS1	–	c.65-3C > T ^b^	120976537	–	12/12, 50/50, 28/33	0	0	0	0	0	–	–	5	[39]
Possibly Pathogenic Variants															
Missense	Exon 18	p.Y1900C	c.5699A > G	121038875	ZP	12/12, 50/50, 36/36	0	0	0	0	0	Probably damaging (0.999)	Deleterious (−6.77)	6	this study
	Exon 20	p.S2017P	c.6049 T > C	121058590	ZP	12/12, 49/49, 36/36	0	0	0	0	0	Probably damaging (0.998)	Neutral (−1.781)	7	this study
Variants of Uncertain Significance															
Missense	Exon 9	p.L940I	c.2818C > A	121000797	ZA (vWFD2-TIL2)	11/12, 49/49, 35/36	0	0	0	0	0	Probably damaging (0.998)	Neutral (0.642)	8	this study
	Exon 10	p.R1033Q	c.3098G > A	121008286	ZA (TIL2)	12/12, 49/50, 31/37	0	0	0.00002	0	0	Possibly damaging (0.704)	Neutral (−0.386)	9	this study
	Exon 13	p.L1439I	c.4315C > A	121028559	ZA (TIL3-vWFD4)	12/12, 49/49, 34/35	0.001	0	0.00078	0.008	0	Probably damaging (0.998)	Neutral (−0.408)	1, 2	[6]
	Exon 14	p.V1646 M	c.4936G > A	121031090	ZA (vWFD4)	12/12, 48/49, 18/37	0	0	0	0.002	0	Benign (0.365)	Neutral (−0.207)	10	this study
	Exon 16	p.P1791R	c.5372C > G	121036081	ZA (vWFD4-ZP)	12/12, 49/49, 20/34	0	0	0.00019	0	0	Benign (0.051)	Neutral (0.259)	3	[4]

^a^Evolutionary conservation in up to 12 primates, 50 mammals, and 38 vertebrates

^b^NNsplice score, 0.95. ZA, zonadhesin-like domain; TIL, three trypsin inhibitor-like repeat; vWFD, von Willebrand factor type D repeat; ZP, zona pellucida domain

### Molecular modeling of novel possibly pathogenic variants in TECTA

**Fig. 3:**
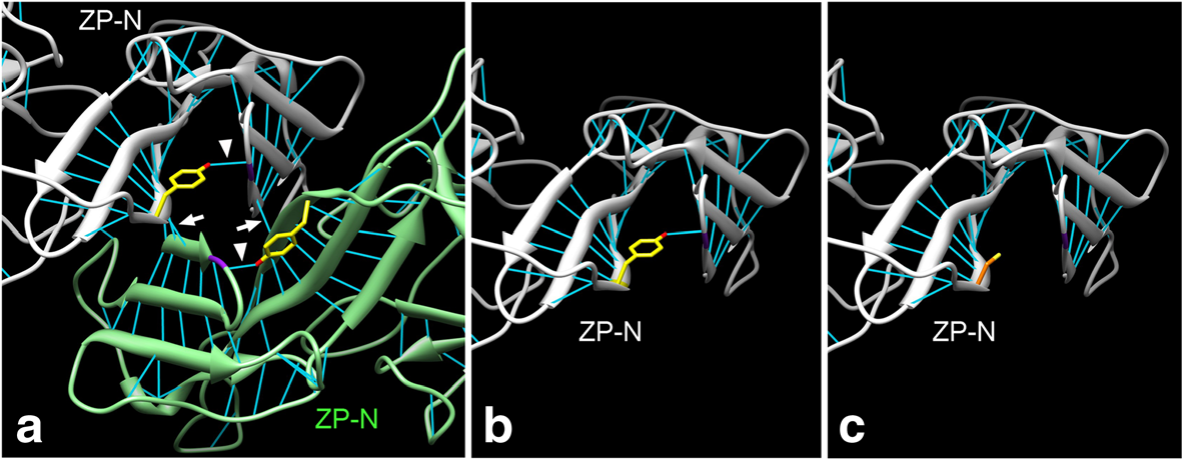
Modeling of possibly pathogenic variants in TECTA. **a** ZP-N dimer structure of TECTA. White and light green colors represent monomers. Light blue lines, hydrogen bonds; yellow, the side chain of Y1900; purple, K1807; arrowheads, hydrogen bonds between Y1900 and K1807; arrows, hydrogen bonds connecting ZP-N domains. **b** Monomer structure of wild type ZP-N. **c** Monomer structure of ZP-N with p.Y1900C mutation. The side chain of C1900 is indicated in orange

Modeling structure of p.Y1900C in TECTA is shown in Fig. 3. The tyrosine at residue 1900 (Y1900) is located in the ZP-N domain, which is involved in dimer formation via hydrogen bonds (arrows in Fig. 3a). Y1900 is predicted to form a hydrogen bond with the lysine at residue 1807 (K1807) (arrowheads in Fig. 3a), to maintain the structure of the dimerization site (Fig. 3a, b). However, in the model of the protein containing the p.Y1900C mutation, this hydrogen bond was absent (Fig. 3c). Hence, the p.Y1900C mutation is likely to disrupt dimerization of the TECTA protein via the ZP-N domain, and result in structural abnormality of the tectorial membrane.

The effect of the p.S2017P mutation, located in the ZP-C domain, was not determined, as no significant alteration in protein structure was predicted after introduction of this mutation in the model; moreover, it is unknown whether this region of the protein is involved in interactions with other molecules.

### Inheritance patterns, clinical features, and audiograms associated with pathogenic or possibly pathogenic variants and VUS in *TECTA*

**Fig. 4:**
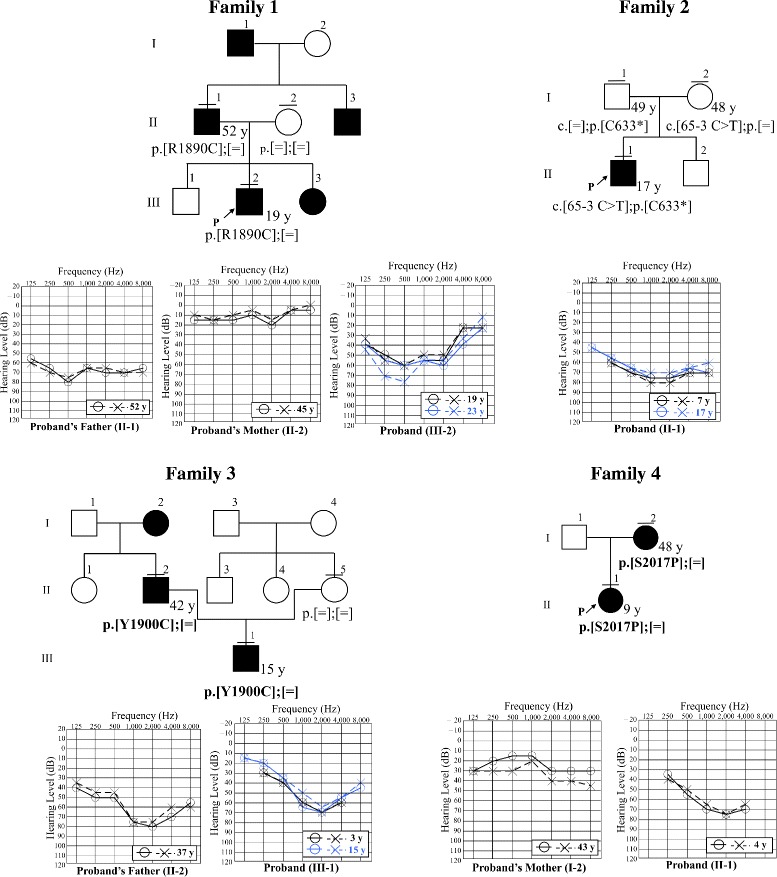
Pedigrees and audiograms of patients with pathogenic and possibly pathogenic *TECTA* variants. P represents the proband. Horizontal bars above circles and squares indicate subjects who underwent genetic testing. Novel variants are presented in bold

Figure 4 shows the pedigrees of four families in which pathogenic or possibly pathogenic variants were identified. Genotyping of the proband and family members, as well as phenotypic characteristics, indicated an AD inheritance pattern in Family 1, Family 3, and Family 4 and an AR inheritance pattern in Family 2. In Family 3 and Family 4, novel possibly pathogenic variants with an AD inheritance pattern were identified. In Family 4, the mother of the proband (I-2) had the possibly pathogenic variant and mild hearing loss, with a flat audiogram, in contrast to the moderate hearing loss and U-shaped audiogram of the proband (II-1). Given this discrepancy, the potential pathogenicity of this variant should be considered with caution.

Clinical data of the four families with pathogenic or possibly pathogenic variants are presented in Table 2. The onset of hearing loss was in the first decade of life in all families. The level of hearing loss was mild to moderate in all cases. All of the probands had shallow U-shaped audiograms. No progression was observed in any case during the follow-up period according to the criteria used in the present study. In Family 1, the most recent audiogram of the father of the proband, who had the mutation, was flat, and the hearing of the proband deteriorated at both high and low frequencies with age, resulting in an audiogram similar to that of the father (Fig. 4). The word recognition score (WRS) was 70–90% in all cases (Additional file 7).

**Table 2:** Clinical data of patients with pathogenic or possibly pathogenic variants

Family	Genotypes	Case	Age of diagnosis	Age at the latest Examination	Audiometric configuration	Hearing loss Severity^a^	Progression^b^	Vestibular symptoms	Inheritance pattern^c^
1	p.[R1890C];[=]	II-1	9 y	52 y	Flat	Moderate	Unknown	Absent	AD
		III-2	7 y	23 y	Shallow U-shaped	Moderate	Stable	Absent	
2	c.[65-3C > T];p.[C633*]	II-1	4 y	17 y	Shallow U-shaped	Moderate	Stable	Absent	AR
3	p.[Y1900C];[=]	II-2	9 y	37 y	U-shaped	Moderate	Unknown	Absent	AD
		III-1	2 y	15 y	Shallow U-shaped	Moderate	Stable	Absent	
4	p.[S2017P];[=]	I-2	Unknown	43 y	Flat	Mild	Unknown	Absent	AD
		II-1	8 m	3 y	Shallow U-shaped	Moderate	Unknown	Absent	

*AD* autosomal dominant; *AR* autosomal recessive

^a^Mild, 20–40 dB HL; moderate, 41–70 dB HL; severe, 71–95 dB HL; profound, >95 dB HL (better hearing ear, averaged over 0.5, 1, 2, and 4 kHz)

^b^Progressive, deterioration of >15 dB HL in the average over the frequencies of 0.5, 1, and 2 kHz within a 10-year period

^c^Inheritance pattern estimated from genotypes and phenotypes of family members

**Fig. 5:**
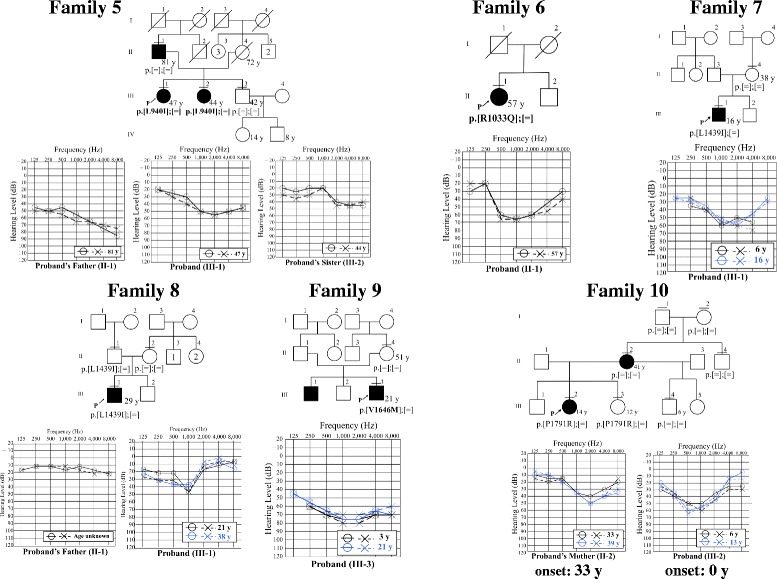
Pedigrees and audiograms of patients with VUS. P represents the proband. Horizontal bars above circles and squares indicate subjects who underwent genetic testing. Novel variants are presented in bold

Figure 5 and Table 3 shows the pedigrees, audiograms, and clinical data of Family 5–10 in which VUS were identified. In Family 7, Family 8 and Family 10, the reported *TECTA* mutations did not show typical family history of AD or did not segregate in family members who were not tested for hearing loss, and/or *TECTA* mutations. It is possible that this may be explained by mild phenotype, de novo mutation, or phenocopy. In Family 7, the parents of the proband (II-3 and II-4) were not known to have hearing loss. Therefore, the mutation may be de novo in the proband. Alternatively, the father (II-3) may carry the mutation and have mild hearing loss that had not been noted. In Family 8, the father (II-1) carried the mutation but did not present with hearing loss. In addition, the allele frequency of this variant in Japanese is high (0.008) for the cause of DFNA8/12. Although the possibility of the low penetrance of this variant cannot be denied, these data suggest that this variant is unlikely to be pathogenic. In Family 10, the mother of the proband (II-2) presented with hearing loss, but did not have the mutation. As the peak frequency of the U-shaped audiogram and the onset of the hearing loss were quite different in the mother from those in the proband (III-2), the cause of hearing loss may be different in the two individuals (suggesting phenocopy), and the mutation in the proband could be de novo or inherited from the father (II-1), who may have the mutation and mild hearing loss that had not been noted. In Family 5, Family 6, and Family 9, novel VUS were identified. In these families, similar to Family 7 and Family 10, individuals reporting normal hearing who had not had hearing tests (Family 5, II-4; Family 6, I-1 and I-2; Family 9, II-1) may have had mild hearing loss. In Family 5, the lack of *TECTA* mutation in the father (of proband, II-1) with hearing loss, suggests that his hearing loss may be due to other causes, such as presbycusis. In Family 6, the mutation in the proband could be de novo.

**Table 3:** Clinical data of patients with VUS

Family	Genotypes	Case	Age of diagnosis	Age at the latest Examination	Audiometric configuration	Hearing lossSeverity^a^	Progression^b^	Vestibularsymptoms	Inheritance pattern^c^
5	p.[L940I];[=]	II-1	40 y	81 y	High-Frequency	Moderate	Unknown	Absent	AD
		III-1	40 y	47 y	Shallow U-shaped	Moderate	Unknown	Absent	
		III-2	33 y	44 y	High-Frequency	Mild	Unknown	Present	
6	p.[R1033Q];[=]	II-1	46 y	57 y	Shallow U-shaped	Moderate	Unknown	Absent	AD
7	p.[L1439I];[=]	III-1	6 y	16 y	Shallow U-shaped	Moderate	Stable	Absent	AD
8	p.[L1439I];[=]	III-1	16 y	38 y	U-shaped	Mild	Stable	Absent	AD
9	p.[V1646 M];[=]	III-1	8 y	23 y	Unknown	Moderate	Unknown	Unknown	AD
		III-3	3 y	21 y	Shallow U-shaped	Moderate	Stable	Absent	
10	p.[P1791R];[=]	III-2	6y	13 y	Shallow U-shaped	Mild	Unknown	Absent	AD

*AD* autosomal dominant; *AR* autosomal recessive

^a^Mild, 20–40 dB HL; moderate, 41–70 dB HL; severe, 71–95 dB HL; profound, >95 dB HL (better hearing ear, averaged over 0.5, 1, 2, and 4 kHz)

^b^Progressive, deterioration of >15 dB HL in the average over the frequencies of 0.5, 1, and 2 kHz within a 10-year period

^c^Inheritance pattern estimated from genotypes and phenotypes of family members

## Discussion

The prevalence of *TECTA* mutations (DFNA8/12) has been investigated in only ADNSHL population, and it was reported as 2.9–4.0% [4, 7]. The present study revealed the prevalence of *TECTA* mutation in patients with MFSNHL for the first time. Pathogenic and possibly pathogenic variants of *TECTA* were found in 4 (6.0%) of 67 patients with bilateral non-syndromic MFSNHL who were prescreened for *GJB2* and m.1555A > G and m.3243A > G mitochondrial DNA mutations. In addition, six patients (9.0%) had VUS, which may underlie their MFSNHL. Among patients with U-shaped audiograms, none had a pathogenic variant and one (2.2%) had VUS. In contrast, four patients (8.7%) with shallow U-shaped audiograms had pathogenic or possibly pathogenic variants and five (10.9%) had VUS. Thus, *TECTA* mutations are likely to be more prevalent among patients with shallow U-shaped audiograms than those with U-shaped audiograms. The frequency of *TECTA* mutations was higher (13.0%) among families which have the family histories compatible with AD than among those which have the family histories compatible with sporadic or AR (2.3%). These conclusions may be limited to the Japanese population because all of the subjects of this study were Japanese. Another limitation of this study is that we have not investigated causes other than *TECTA* and we cannot deny the possibility that other causes might have effected on hearing of the present patients. However, the significance of the *TECTA* variants would not be likely to be reversed solely by such effects, since we evaluated these variants with the strictly established criteria.

Regarding genotype-phenotype correlation, three pathogenic and possibly pathogenic AD variants (p.R1890C, p.Y1900C, p.S2017P) were located in the ZP domain (Fig. 1). This correlation between ZP domain mutations and MFSNHL is consistent with previous studies [16, 17]. In one AR family, identified pathogenic variants (p.C633*, and c.65-3C > T) were truncating mutations and the moderate U-shaped hearing loss was present. This correlation between AR truncating mutations in any regions and MFSNHL is also consistent with previous studies [10, 14, 15]. Five VUS of AD inheritance patterns were located in the ZA domain or between ZA and ZP domain, two of which have been reported previously [4, 6], indicating that regions other than the ZP domain may also be associated with MFSNHL. Since novel *TECTA* mutations keep increasing [33, 34], genotype-phenotype correlations may be expanded along with the progress of genetic studies. Because two previously reported mutations, p.L1439I and p.P1791R, were regarded as VUS in the present study, pathogenicity of each variants needs to be validated with caution.

Regarding the clinical features of hearing loss related to *TECTA* mutations, all patients with mutations had good WRSs and all the patients whose audiograms could be evaluated for more than 10 years showed non-progressive hearing loss. It was previously reported that WRSs in patients with *TECTA* mutations are superior to those in individuals with age-related hearing impairment presenting with the same levels of hearing loss [17]. This phenomenon was explained by the fact that thresholds are maintained at high frequencies [17], and the lack of primary damage to the function of hair cells and cochlear nerves in *TECTA* patients may also be relevant. Patients with cysteine-replacing mutations in *TECTA* exhibit progressive hearing loss, while those with other mutations have non-progressive symptoms [16, 17]. None of the mutations in the present study were cysteine-replacing, and none of the patients had progressive hearing loss; therefore, our data are in agreement with the findings of previous studies.

The present study revealed that more than 90% of non-syndromic MFSNHL is likely caused by factors other than *TECTA* mutations. Thus, there may be few unknown causative genes for MFSNHL with high prevalence, or many other causative genes with low prevalence. Other than *TECTA*, three genes, *COL11A2* (DFNA13, DFNB53) [35, 36], *EYA4* (DFNA10) [37], and *CCDC50* (DFNA44) [38], are reported to cause MFSNHL. The frequency of mutations in these genes and other unknown causative genes could be determined by comprehensive genetic testing using next generation sequencing in patients with MFSNHL in future investigations.

## Conclusions

*TECTA* gene mutations were identified in 6.0% of MFSNHL and were more frequent in cases with shallow U-shaped audiograms than those with U-shaped audiograms. Two novel possibly pathogenic variants were identified and two previously reported mutations were considered as VUS. The frequency of *TECTA* mutations was higher in families with the family history compatible with AD than in those which have the family history compatible with sporadic or AR.

## Additional files

Additional file 1: Methods of screening for *GJB2* mutations and mitochondrial m.1555A > G and m.3243A > G mutations. Methods of screening for GJB2 mutation and mitochondrial m.1555A > G and m.3243A > G mutations used in this study. (DOCX 15 kb)

Additional file 2: Primers for *TECTA* sequencing used in this study. A list of oligonucleotide primers for *TECTA* sequencing used in this study. (XLSX 14 kb)

Additional file 3: A breakdown list of species used for evaluation of amino acid conservation. A table describing the breakdown list of species used for evaluation of amino acid conservation in this study. (XLSX 14 kb)

Additional file 4: The number of patients with variants classified by pathogenicity in two types of audiograms. A table describing the number of patients with variants classified by pathogenicity in patients with U-shaped audiogram and shallow U-shaped audiogram among all the patients tested for *TECTA*. (XLSX 8 kb)

Additional file 5: Non-pathogenic variants identified in this study. A table describing non-pathogeni variants identified in this study. (XLSX 21 kb)

Additional file 6: Chromatograms of DNA sequences of patients with pathogenic variants, possibly pathogenic variants, and VUS. Description of data: A figure showing partial chromatograms of DNA sequences of patients with pathogenic variants, possibly pathogenic variants, and VUS. (PPTX 3398 kb)

Additional file 7: Speech audiograms of patients with pathogenic and possibly pathogenic variants. A figure showing speech audiograms of patients with pathogenic and possibly pathogenic variants. (PPTX 268 kb)

## References

[1] Smith RJ, Bale JF, White KR (2005). Sensorineural hearing loss in children. Lancet.

[2] Van Camp G, Smith RJH. Hereditary Hearing Loss Homepage. http://hereditaryhearingloss.org. Accessed May 8th 2017.

[3] Xia W, Liu F, Ma D (2016). Research progress in pathogenic genes of hereditary non-syndromic mid-frequency deafness. Front Med.

[4] Hildebrand MS, Morin M, Meyer NC, Mayo F, Modamio-Hoybjor S, Mencia A (2011). DFNA8/12 Caused by TECTA mutations is the most identified subtype of nonsyndromic autosomal dominant hearing loss. Hum Mutat.

[5] Kim AR, Chang MY, Koo JW, Oh SH, Choi BY (2015). Novel TECTA mutations identified in stable sensorineural hearing loss and their clinical implications. Audiol Neurootol.

[6] Miyagawa M, Naito T, Nishio SY, Kamatani N, Usami S (2013). Targeted exon sequencing successfully discovers rare causative genes and clarifies the molecular epidemiology of Japanese deafness patients. PLoS One.

[7] Moteki H, Nishio SY, Hashimoto S, Takumi Y, Iwasaki S, Takeichi N (2012). TECTA mutations in Japanese with mid-frequency hearing loss affected by zona pellucida domain protein secretion. J Hum Genet.

[8] Verhoeven K, Van Laer L, Kirschhofer K, Legan PK, Hughes DC, Schatteman I (1998). Mutations in the human alpha-tectorin gene cause autosomal dominant non-syndromic hearing impairment. Nat Genet.

[9] UniProt. http://www.uniprot.org. Accessed 26 July 2017.

[10] Mustapha M, Weil D, Chardenoux S, Elias S, El-Zir E, Beckmann JS (1999). An alpha-tectorin gene defect causes a newly identified autosomal recessive form of sensorineural pre-lingual non-syndromic deafness, DFNB21. Hum Mol Genet.

[11] Legan PK, Lukashkina VA, Goodyear RJ, Lukashkin AN, Verhoeven K, Van Camp G (2005). A deafness mutation isolates a second role for the tectorial membrane in hearing. Nat Neurosci.

[12] Legan PK, Goodyear RJ, Morin M, Mencia A, Pollard H, Olavarrieta L (2014). Three deaf mice: mouse models for TECTA-based human hereditary deafness reveal domain-specific structural phenotypes in the tectorial membrane. Hum Mol Genet.

[13] Xia A, Gao SS, Yuan T, Osborn A, Bress A, Pfister M (2010). Deficient forward transduction and enhanced reverse transduction in the alpha tectorin C1509G human hearing loss mutation. Dis Model Mech.

[14] Meyer NC, Alasti F, Nishimura CJ, Imanirad P, Kahrizi K, Riazalhosseini Y (2007). Identification of three novel TECTA mutations in Iranian families with autosomal recessive nonsyndromic hearing impairment at the DFNB21 locus. Am J Med Genet A.

[15] Naz S, Alasti F, Mowjoodi A, Riazuddin S, Sanati MH, Friedman TB (2003). Distinctive audiometric profile associated with DFNB21 alleles of TECTA. J Med Genet.

[16] Balciuniene J, Dahl N, Jalonen P, Verhoeven K, Van Camp G, Borg E (1999). Alpha-tectorin involvement in hearing disabilities: one gene--two phenotypes. Hum Genet.

[17] Plantinga RF, de Brouwer AP, Huygen PL, Kunst HP, Kremer H, Cremers CW (2006). A novel TECTA mutation in a Dutch DFNA8/12 family confirms genotype-phenotype correlation. J Assoc Res Otolaryngol.

[18] NNSPLICE. http://www.fruitfly.org/seq_tools/splice.html. Accessed 15 Aug 2016.

[19] 1000 Genomes. http://www.1000genomes.org. Accessed 15 Aug 2016.

[20] NHLBI Esome Sequencing Project (ESP). http://evs.gs.washington.edu/EVS. Accessed 15 Aug 2016.

[21] ExAC Browser (Beta). http://exac.broadinstitute.org. Accessed 15 Aug 2016.

[22] Human Genetic Variation Database. http://www.hgvd.genome.med.kyoto-u.ac.jp/. Accessed 15 Aug 2016.

[23] Integrative Japanese Genome Variation Database. https://ijgvd.megabank.tohoku.ac.jp. Accessed 15 Aug 2016.

[24] UCSC Genome Browser. http://genome.ucsc.edu/index.html. Accessed 15 Aug 2016.

[25] PolyPhen-2. http://genetics.bwh.harvard.edu/pph2. Accessed 15 Aug 2016.

[26] PROVEAN. http://provean.jcvi.org/index.php. Accessed 15 Aug 2016.

[27] Arnold K, Bordoli L, Kopp J, Schwede T (2006). The SWISS-MODEL workspace: a web-based environment for protein structure homology modelling. Bioinformatics.

[28] Kiefer F, Arnold K, Kunzli M, Bordoli L, Schwede T (2009). The SWISS-MODEL repository and associated resources. Nucleic Acids Res.

[29] Han L, Monne M, Okumura H, Schwend T, Cherry AL, Flot D (2010). Insights into egg coat assembly and egg-sperm interaction from the X-ray structure of full-length ZP3. Cell.

[30] Bokhove M, Nishimura K, Brunati M, Han L, de Sanctis D, Rampoldi L (2016). A structured interdomain linker directs self-polymerization of human uromodulin. Proc Natl Acad Sci U S A.

[31] Monne M, Han L, Schwend T, Burendahl S, Jovine L (2008). Crystal structure of the ZP-N domain of ZP3 reveals the core fold of animal egg coats. Nature.

[32] Pettersen EF, Goddard TD, Huang CC, Couch GS, Greenblatt DM, Meng EC (2004). UCSF chimera--a visualization system for exploratory research and analysis. J Comput Chem.

[33] Asgharzade S, Tabatabaiefar MA, Modarressi MH, Ghahremani MH, Reiisi S, Tahmasebi P (2017). A novel TECTA mutation causes ARNSHL. Int J Pediatr Otorhinolaryngol.

[34] Gürtler N, Röthlisberger B, Ludin K, Schlegel C, Lalwani AK (2017). The Application of Next-Generation Sequencing for Mutation Detection in Autosomal-Dominant. Hereditary Hearing Impairment. Otol Neurotol.

[35] Chen W, Kahrizi K, Meyer NC, Riazalhosseini Y, Van Camp G, Najmabadi H (2005). Mutation of COL11A2 causes autosomal recessive non-syndromic hearing loss at the DFNB53 locus. J Med Genet.

[36] McGuirt WT, Prasad SD, Griffith AJ, Kunst HP, Green GE, Shpargel KB (1999). Mutations in COL11A2 cause non-syndromic hearing loss (DFNA13). Nat Genet.

[37] van Beelen E, Oonk AM, Leijendeckers JM, Hoefsloot EH, Pennings RJ, Feenstra I (2016). Audiometric characteristics of a Dutch DFNA10 family with mid-frequency hearing impairment. Ear Hear.

[38] Modamio-Hoybjor S, Mencia A, Goodyear R, del Castillo I, Richardson G, Moreno F (2007). A mutation in CCDC50, a gene encoding an effector of epidermal growth factor-mediated cell signaling, causes progressive hearing loss. Am J Hum Genet.

[39] Moteki H, Azaiez H, Booth KT, Shearer AE, Sloan CM, Kolbe DL, et al. Comprehensive genetic testing with ethnic-specific filtering by allele frequency in a Japanese hearing-loss population. Clin Genet. 2015; doi:10.1111/cge.12677. 10.1111/cge.12677 PMC4783301 26346818

