# Supplementary material for: Prevalence of TECTA mutation in patients with mid-frequency sensorineural hearing loss
Source: Orphanet J Rare Dis. 2017 Sep 25;12:157. doi: 10.1186/s13023-017-0708-z (PMC5613382; doi:10.1186/s13023-017-0708-z)
Supplement: Supplementary file 1 — Methods of screening for GJB2 mutations and mitochondrial m.1555A > G and m.3243A > G mutations. Methods of screening for GJB2 mutation and mitochondrial m.1555A > G and m.3243A > G mutations used in this study. (DOCX 15 kb) [file 13023_2017_708_MOESM1_ESM.docx]

Additional file 1. Methods of screening for GJB2 mutation and mitochondrial m.1555A>G and m.3243A>G mutations.

Screening for *GJB2* mutation was performed by polymerase chain reaction (PCR) using the primer set: 5’-CCTATGACAAACTAAGTTGGTTC-3’ and 5’-TGAGCACGGGTTGCCTC-3’. PCR conditions were as follows: 5 min denaturation at 95°C; 35 cycles of 95°C for 1 min, 57.3°C for 1 min, and 72°C for 2 min; followed by 72°C for 2 min, and ending with a holding period at 4°C. Reactions were performed in a PC818 thermal cycler (ASTEC, Japan). PCR products were purified and subjected to Sanger sequencing using a BigDye Terminator v3.1 Cycle Sequencing Kit and a 3730 DNA Analyzer (Applied Biosystems, USA).

Screening for the mitochondrial m.1555A>G and m.3243A>G mutations was performed by restriction fragment length polymorphism (RFLP) analysis. To detect the m.1555A>G mutation, PCR was carried out using the primer set: 5’-GTAAAGACGTTAGGTCAAGG-3’ and 5’-TACATAGACGGGTGTGCTCTT-3’. PCR conditions were as follows: 5 min denaturation at 95°C; 30 cycles of 94°C for 1 min, 58°C for 1 min, and 72°C for 1 min; followed by 72°C for 1 min, and ending with a holding period at 4°C. PCR products were incubated with the restriction enzyme, BsmAI (Takara Bio, Japan) and then subjected to electrophoresis on agarose gels. Due to loss of the BsmAI site, the PCR fragment including the mutation was visible as a single uncut band on electrophoresis, whereas the normal PCR fragments were digested into two fragments. To detect the m.3243A>G mutation, PCR was carried out using the primer set: 5’-AAGGTTCGTTTGTTCAACGA-3’ and 5’-AGCGAAGGGTTGTAGTAGCC-3’ under the same conditions as those for m.1555A>G. PCR products were incubated with the restriction enzyme ApaI (New England BioLabs, USA) and then subjected to agarose gel electrophoresis. Due to the creation of an ApaI site by the m.3243A>G alteration, PCR fragments containing the mutation were digested into two fragments on electrophoresis, whereas the normal PCR fragments was single. All experiments included positive controls.
