# Supplementary figures and images for: Prevalence of TECTA mutation in patients with mid-frequency sensorineural hearing loss
Source: Orphanet J Rare Dis. 2017 Sep 25;12:157. doi: 10.1186/s13023-017-0708-z (PMC5613382; doi:10.1186/s13023-017-0708-z)

## Slide 1
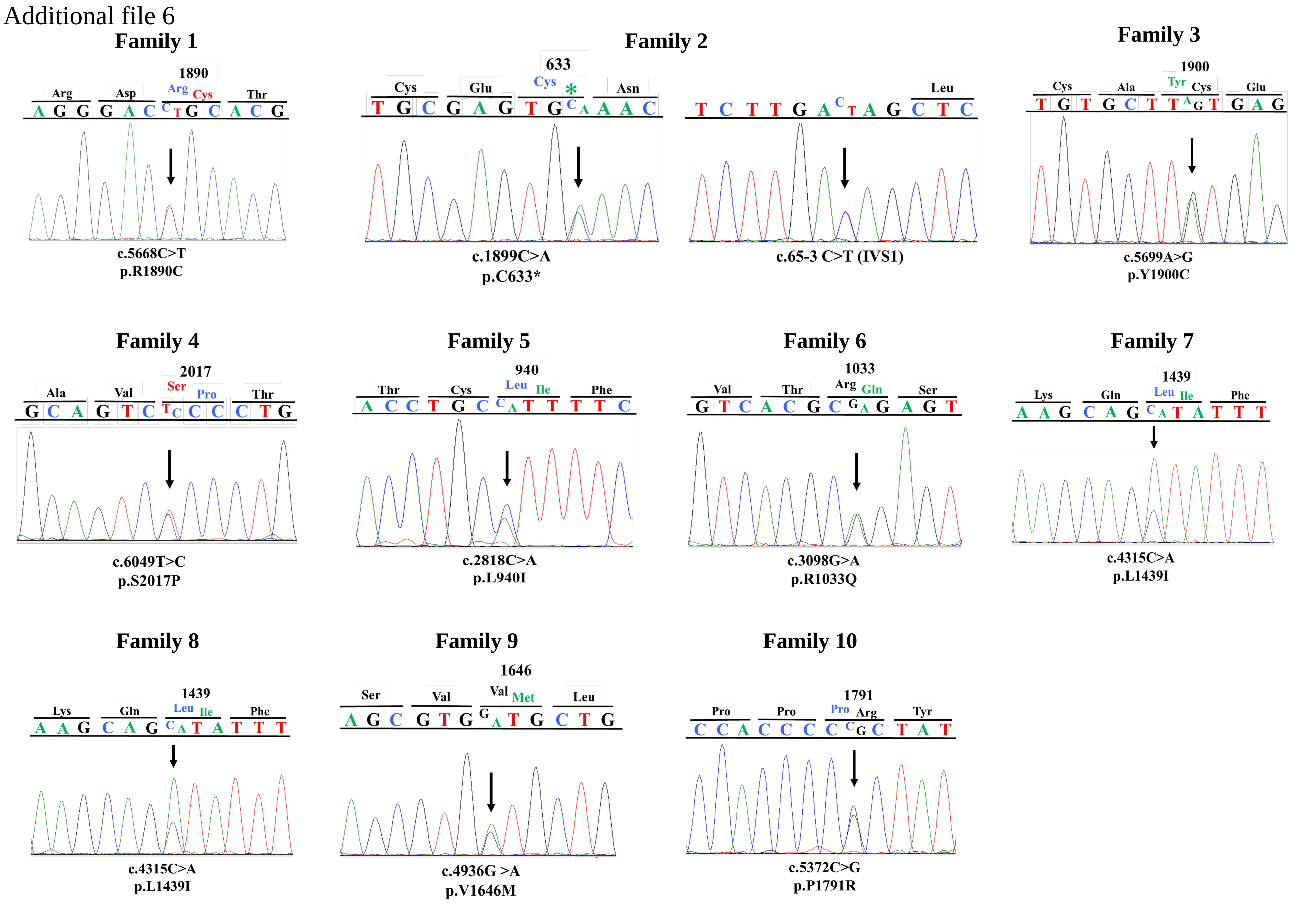

Additional file 6
Family 3
Family 1
Family 2
Family 4
Family 5
Family 6
Family 7
Family 8
Family 9
Family 10

Supplement: Supplementary file 6 — Chromatograms of DNA sequences of patients with pathogenic variants, possibly pathogenic variants, and VUS. Description of data: A figure showing partial chromatograms of DNA sequences of patients with pathogenic variants, possibly pathogenic variants, and VUS. (PPTX 3398 kb) [file 13023_2017_708_MOESM6_ESM.pptx]

## Slide 1
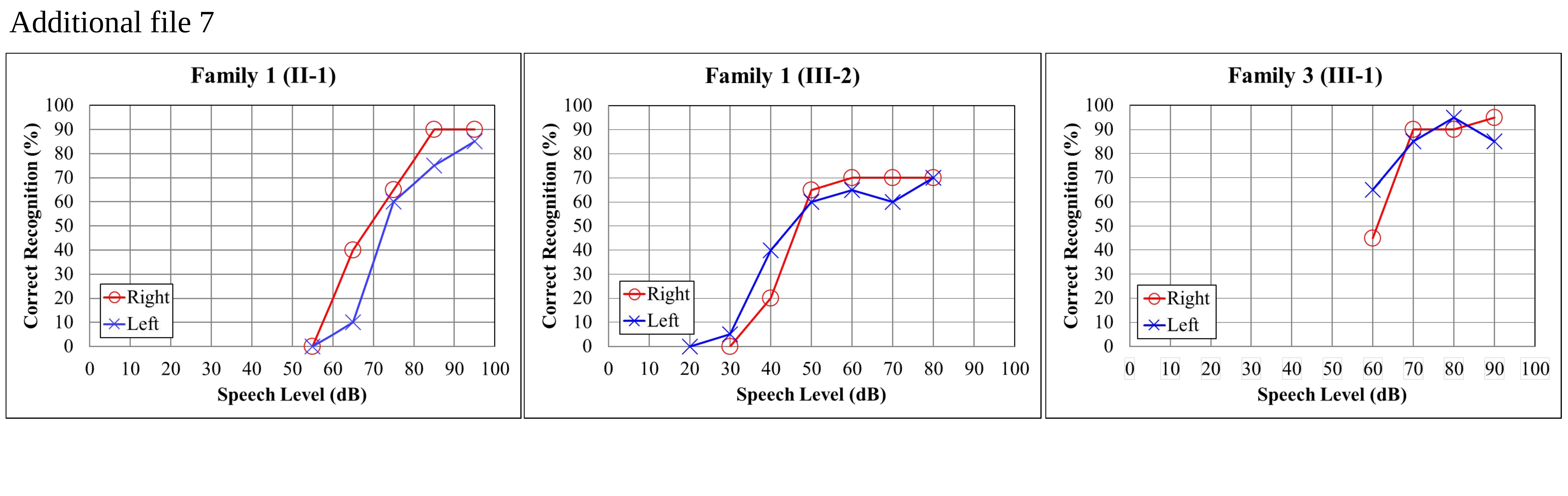

Additional file 7

Supplement: Supplementary file 7 — Speech audiograms of patients with pathogenic and possibly pathogenic variants. A figure showing speech audiograms of patients with pathogenic and possibly pathogenic variants. (PPTX 268 kb) [file 13023_2017_708_MOESM7_ESM.pptx]
